# CD169 Expression on Lymph Node Macrophages Predicts in Patients With Gastric Cancer

**DOI:** 10.3389/fonc.2021.636751

**Published:** 2021-03-19

**Authors:** Keiichiro Kumamoto, Takashi Tasaki, Koji Ohnishi, Michihiko Shibata, Shohei Shimajiri, Masaru Harada, Yoshihiro Komohara, Toshiyuki Nakayama

**Affiliations:** ^1^ Department of Pathology, University of Environmental and Occupational Health, Fukuoka, Japan; ^2^ Third Department of Internal Medicine, University of Occupational and Environmental Health, Fukuoka, Japan; ^3^ Department of Pathology, Graduate School of Medical and Dental Sciences, Kagoshima University, Kagoshima, Japan; ^4^ Department of Cell Pathology, Graduate School of Medical Sciences, Kumamoto University, Kumamoto, Japan; ^5^ Center for Metabolic Regulation of Healthy Aging, Kumamoto University, Kumamoto, Japan

**Keywords:** lymph node, macrophage, CD169, gastric cancer, lymphocyte

## Abstract

The induction of an anti-cancer immune responses is potentially associated with the efficacy of anti-cancer therapy. Recent studies have indicated that sinus macrophages in regional lymph nodes are involved in anti-cancer immune responses in the cancer microenvironment. In the present study, we investigated the correlation between lymphocyte infiltration in cancer tissues and macrophage activation in regional lymph nodes. We retrospectively identified 294 patients with gastric cancer who underwent surgery from 2008 to 2012. Using immunohistochemistry, we evaluated CD169-expression on CD68-positive macrophages, and the density of CD8-postive lymphocytes in tumor microenvironment. We statistically examined the correlation between CD169 and CD8 expression, and performed Cox regression analysis of potential prognostic factors, including CD169 and CD8 expression, for cancer-specific survival (CSS) in patients with total and advanced gastric cancer. CD169 overexpression in lymph node sinus macrophages (LySMs) was positively correlated to the density of CD8-positive lymphocytes in primary cancer tissues (R = 0.367, *p* < 0.001). A high density of CD8-positive T lymphocytes in the primary site and a high level of CD169 expression in LySMs were independently associated with greater CSS in patients with total and advanced gastric cancer (*p* < 0.05 for all). The expression on CD169 in LySMs is a predictor of a favorable clinical course in patients with gastric cancer, and might be useful for evaluating anti-cancer immune responses.

## Introduction

Gastric cancer is one of the most common cancers, with about 865,000 patients with this disease dying worldwide each year (1). Various treatments including cancer immunotherapies have been used to treat gastric cancer, but the prognosis remains poor. Recently, immune checkpoint inhibitors have been approved to treat patients with gastric cancer. Tumor-infiltrating lymphocytes (TILs) are involved in anti-cancer immune responses with a high density of such cells in cancer tissues associated with a favorable prognosis in various cancers, including gastric cancer (2–5). A high density of TILs in cancer tissues is suggested to be associated with a better clinical effect of chemotherapy in patients with advanced gastric cancer (6). Thus, the induction of an anti-cancer immune responses is necessary to improve the efficacy of anti-cancer therapy.

Lymph nodes, as immune organs, play an important role in the induction of specific immune responses to cancer (7, 8). Various antigens from peripheral tissues flow into lymph nodes, where dendritic cells and macrophages act as antigen-presenting cells (9, 10). It is well-known that dendritic cells have strong antigen-presenting ability. In addition to dendritic cells, lymph node sinus macrophages (LySMs) have also been suggested to have antigen-presenting capacity in animal studies.

CD169, also called sialoadhesin, is the foremost member of the sialic acid-binding lectin (Siglec) superfamily (7). It binds sialylated glycoproteins including CD43 (sialophorin) and MUC1 and is involved in cell–cell adhesion as well as cell–pathogen interactions (11–14). CD169 expression is found in splenic marginal metallophilic macrophages and in certain tissue macrophages in bone marrow, colon, liver, and lung, as well as in LySMs (11, 15). CD169-positive macrophages express both M1- and M2-related genes, and are considered as a unique subset that differ from M1/M2-like macrophages (16, 17). CD169-positive LySMs were involved in antigen presentation and the induction of cytotoxic T lymphocytes, in addition to dendritic cells, in a mouse model (18, 19). The downregulation of CD169 in pre-metastatic regional lymph nodes was associated with lymph node metastasis in a rat model (20). In histopathological studies using human resected samples, a correlation between a high CD169 expression level in LySMs and a favorable clinical course has been reported in several cancers including colorectal cancer (21). A correlation between anti-cancer immune responses and a high level of CD169 expression has also been suggested in these tumors. These findings indicated that LySMs in regional lymph nodes are closely associated with anti-cancer immune responses since they engulf dead cells and debris from tumor tissues (22). However, the direct mechanisms underlying the role of CD169 in the induction of immune responses have not yet been clarified. Because CD169 expression is up-regulated by type I interferons, CD169 expression has been considered a surrogate marker of active immune responses in lymph nodes (23).

No studies have yet examined the role of LySMs in patients with gastric cancer. In the present study, we aimed to elucidate the association between CD169 LySMs and prognosis in gastric cancer, including its pathological stage and subgroups: histology, tumor-stroma ratio, distant metastasis, and LN metastasis. In order to determine the significance of CD169 expression in patients with gastric cancer, we used tissue specimens to investigate the correlation between CD169 expression on LySMs and CD8^+^ T-cell infiltration in primary lesions. We also examined the relationship between CD169 expression and various clinicopathological factors.

## Materials and Methods

### Samples

We conducted a retrospective analysis in accordance with the Declaration of Helsinki and with the approval of the Hospital of the University of Environmental and Occupational Health (UOEH), and Wakamatsu Hospital of UOEH (H30-172). The present study utilized paraffin-embedded specimens of primary lesions and regional lymph nodes (RLNs) resected from 294 patients with gastric cancer who had undergone surgery at the Hospital of the UOEH from 2008 to 2012 and Wakamatsu Hospital of the UOEH from 2011 to 2012. We excluded patients who died from non-primary cancer causes or patients who were no longer available for follow-up within a year after surgery. We also excluded cases in which the lymph nodes were difficult to evaluate and in which no lymph node resection had been performed.

### Histology

Samples from 294 gastric cancer cases were stained with hematoxylin and eosin (HE) and evaluated histopathologically by two or three pathologists (KK, TT, and TN) who were blinded to clinical outcomes. As previously published, the tumor-stroma ratio (TSR) was calculated as the percentage of stroma relative to tumor area; tumors were subgrouped as having a high (>50%) or low TSR (24–26). We selected the most invasive tumor area (0.25 -0.50 mm^2^ total area) for TSR evaluation, and excluded areas with necrosis or mucin deposition. TSR evaluation was also conducted by two or three pathologists (KK, TT, and TN) who were blinded to clinical outcomes.

### Immunohistochemistry

Tumor tissues and RLNs were fixed in 10% neutral formalin and embedded in paraffin. Anti-CD169 (clone HSn 7D2; Santa Cruz Biotechnology, CA, USA), anti- CD68 (clone PG-M; Agilent Technologies, CA, USA), and anti-CD8 (clone C8/144B; Nichirei, Tokyo, Japan) antibodies were used as primary antibodies for immunohistochemistry (IHC). Antigen retrieval and IHC were performed as previously published (27). Lymph nodes without metastasis were used as controls for CD68 and CD169 expressions. For counting CD169^+^ and CD68^+^ cells in RLNs and CD8^+^ cells in tumors, we used the HALO 2.3 system (Indica Labs, Albuquerque, NM, USA). We selected four random fields (0.25 mm^2^ per field, total 1.00 mm^2^) from the primary tumor to count CD8^+^ T cells. To count CD68^+^ and CD169^+^ macrophages in RLNs, we delineated the RLN sinus with a line in two to four fields (0.10 to 0.25 mm^2^ per field, total 0.50 mm^2^) in serial sections. After counting cells and measuring these areas, we calculated the density of CD8^+^ T cell infiltration into the tumor, and the ratio of CD169^+^ cells in CD68^+^ LySMs. For double-IHC, HistoGreen substrate (green color; AYS-E109, Eurobio Scientific, Les Ulis, France) was used for peroxidase-based immunostaining.

### Statistical Analysis

We carried out statistical analyses using SPSS 25 software (IBM, Chicago, IL, USA). Bivariate comparisons of clinicopathological features between patients with high (n = 135) and low (n = 159) ratios of CD169^+^ to CD68^+^ LySMs were performed using a χ^2^-test. The relationship between two numerical factors was analyzed using Spearman’s correlation analysis. The association of multiple prognostic factors with cancer-specific survival was assessed using univariate and multivariate Cox proportional hazard model analysis. Multivariate analysis included age, sex, histology, depth of invasion, LN metastasis, distant metastasis, lymphatic invasion, vascular invasion, tumor-stroma ratio, density of CD8^+^ T cells, and the ratio of CD169^+^ to CD68^+^ LySMs. Survival curves were calculated using the Kaplan-Meier method, and the difference between survival curves was analyzed using the log-rank test. Differences were considered statistically significant at *P*-values of < 0.05.

## Results

### CD169 Expression in LySMs Was Significantly Associated With the Density of Infiltrating CD8^+^ T Cells in Primary Cancer Lesion

**Figure 1:**
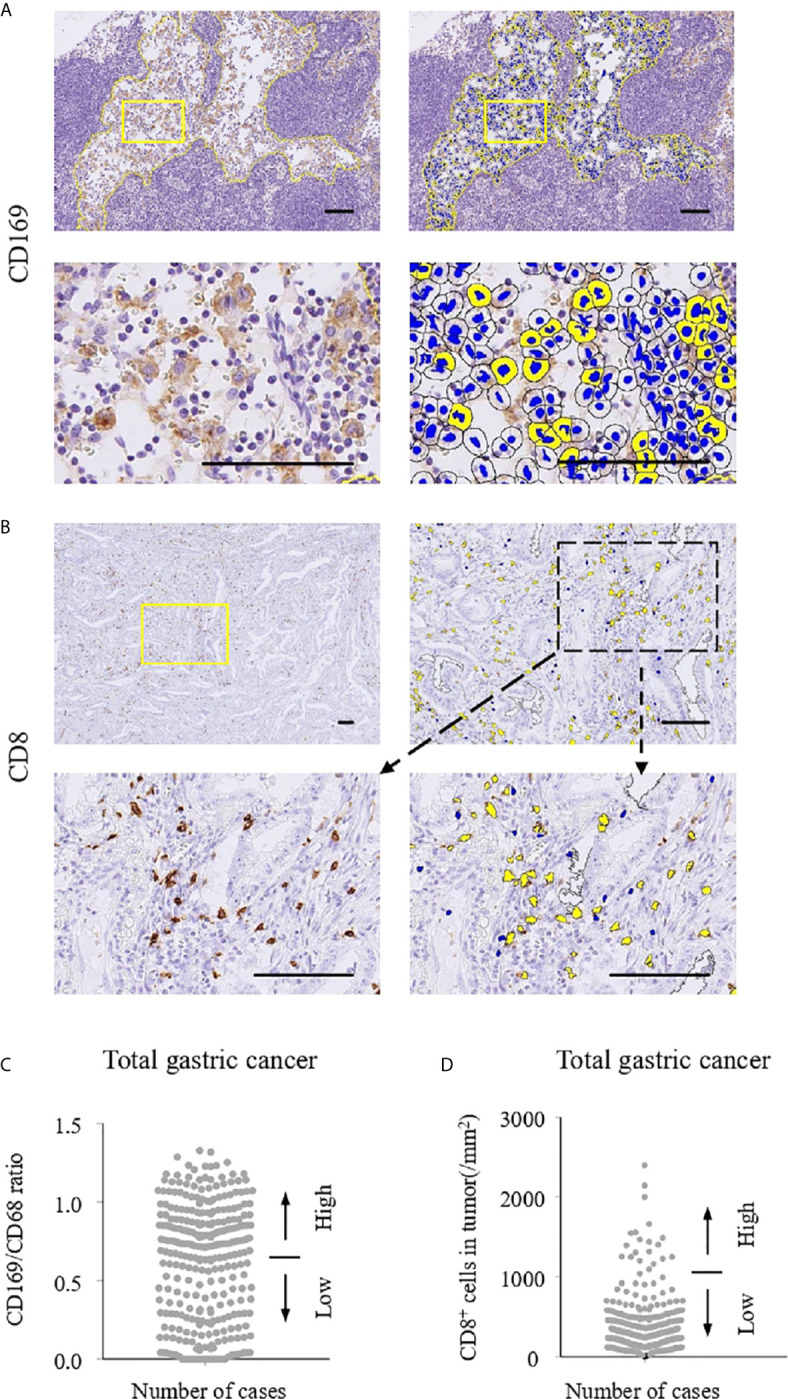
Immunohistochemistry and cell counting system. The numbers of CD169^+^ and CD68^+^ cells in regional lymph nodes (RLNs) and CD8^+^ cells in tumors were evaluated using HALO 2.3 as described in Materials and Methods. Scale bar = 100 μm. Representative immunohistochemistry (IHC)**** stains of CD169 **(A)** and CD8 **(B)** are shown. Positive cells in selected areas surrounded by yellow lines were counted automatically by HALO 2.3. **(C)** Number of CD169/CD68 ratio in lymph node sinus macrophages (LySMs). **(D)** Number of CD8 expressions in primary tumor. Patients were divided into two groups according to their CD169/CD68 ratio: < 0.65 was defined as low and ≥ 0.65 was defined as high. With regard to CD8^+^ cells, the patients were divided into two groups according to cell density: < 287/mm^2^ was defined as low and ≥ 287/mm^2^ was defined as high.

**Figure 2:**
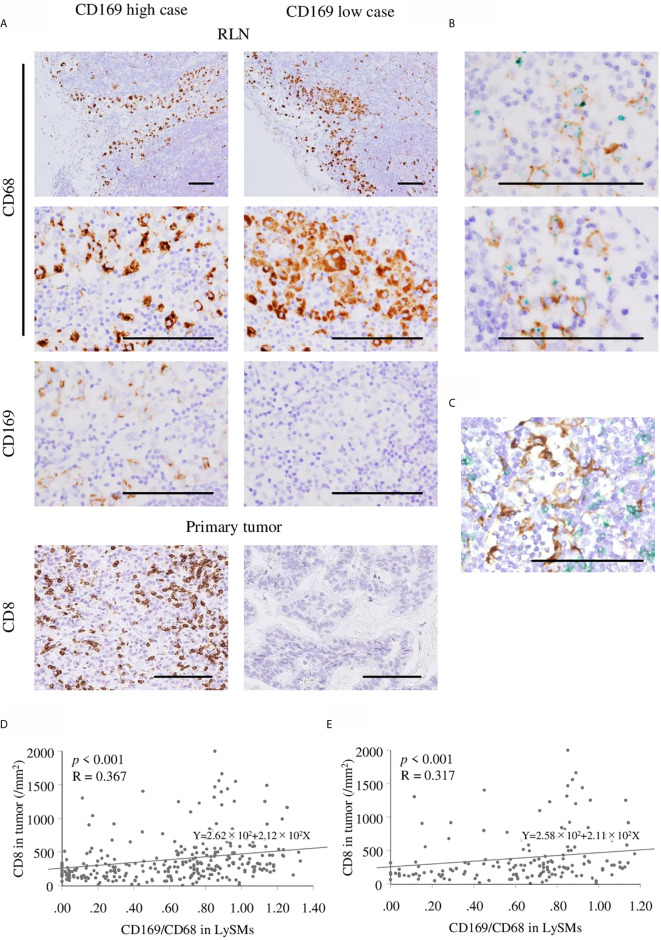
Immunohistochemistry of CD169^+^ and CD68^+^ macrophage in regional lymph nodes (RLN), and CD8^+^ cells in primary tumor. Scale bar = 100 μm. **(A)** Representative figures of immunohistochemistry (IHC) images from CD169 high and low cases are shown. Lymph node sinus macrophages (LySMs) were positive for CD68 in both two patients, although, CD169 expression differed. High infiltration of CD8^+^ T cells in primary tumor tissues was seen in a CD169^high^ case and low infiltration of CD8^+^ T cells in primary tumor tissues was seen in a CD169^low^ case. **(B)** Double IHC of CD68(green) and CD169(brown) showed CD169 was expressed on CD68-positive macrophages. Correlation between the number of CD8^+^ T cells in primary tumor tissues and CD169/CD68 ratio in LySMs were tested by Spearman’s correlation test. **(C)** Double IHC of CD8(green) and CD169(brown) showed the direct cell-cell interaction between LySM and T cells in sinus area. Scatter plots of total **(D)** and in advanced **(E)** gastric cancer cases were shown. RLN, regional lymph node.

Immunohistochemistry for CD68 and CD169 was performed using RLN specimens, and that for CD8 was done using primary cancer specimens in 294 gastric cancer cases. The density of positive cells was evaluated by the HALO 2.3 system as described in the Materials and Methods section (**Figures 1A, B**). As shown in **Figure 1**, the density of CD8-positive cells ranged from 8.33 to 2399.26 cells/mm^2^ (mean, 394.33 cells/mm^2^; median, 286.81 cells/mm^2^). The ratio of CD169^+^ to CD68^+^ cells ranged from 0.00% to 132.78% (mean, 62.14%; median, 71.38%; **Figure 1D**). Representative IHC images of samples of two patients are shown in **Figure 2**. Since it is well known that CD169 expression was restricted in CD68^+^ LySMs in the lymph node, the expression on CD169 in LySMs was evaluated as the ratio of CD169^+^ to CD68^+^ cells (**Figure 2B**). The direct cell-cell interaction between CD169^+^ LySM and CD8^+^ TIL was detected in sinus area of lymph node (**Figure 2C**). Next, we tested the correlation between CD8^+^ TILs in tumor tissues and CD169 expression in LySM. We found that the ratio of CD169^+^ to CD68^+^ cells was positively correlated to the density of CD8^+^ TILs in both total and advanced gastric cancer (total; R = 0.367, *p* < 0.001; advanced; R = 0.317, *p* < 0.001; **Figures 2D, E**). These observations indicated a significant correlation between high CD169 expression in LySMs and high immune responses in the tumor microenvironment.

### High CD169 Expression in LySMs and a High Density of Infiltrating CD8^+^ T Cells in Primary Cancer Lesion Were Associated With a Favorable Clinical Course

**Figure 3:**
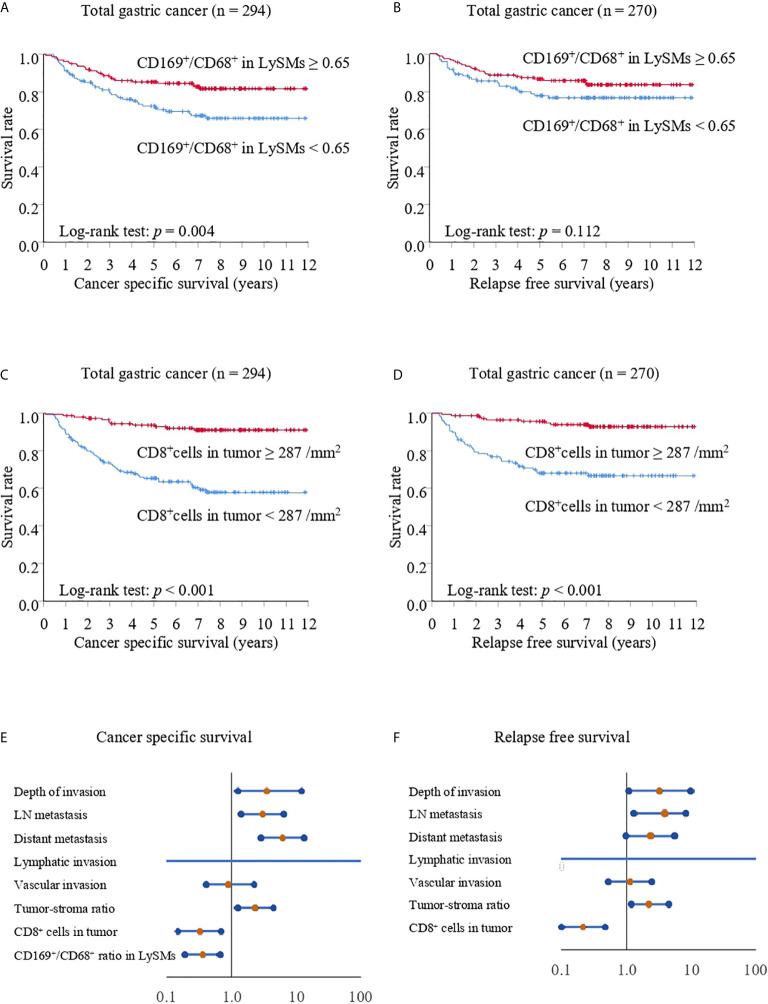
Kaplan-Meier cancer-specific survival and relapse-free survival curves for total gastric cancer cases. The patients were divided into two groups according to their CD169/CD68 ratio: < 0.65 was defined as low and ≥ 0.65 was defined as high **(A, B)** or divided into two groups according to their density of CD8^+^ cells: < 287/mm^2^ was defined as low and ≥ 287/mm^2^ was defined as high **(C, D)**. **(A, C)** Cancer-specific survival (CSS) curves for patients with total gastric cancer. **(B, D)** Relapse-free survival (RFS) curves for patients with total gastric cancer. **(E)** Forest plot analysis of CSS. **(F)** Forest plot analysis of RFS.

Patients were divided into CD169^low^ and CD169^high^ groups, with the cut-off value set to 65% (**Figure 1**) and the relationship with clinicopathological features analyzed. The expression on CD169 in LySMs was not associated with age, sex, histological subtype, depth of invasion, lymphovascular invasions or metastasis (**Table 1**). Next, we tested the correlation between cancer-specific overall survival time (CSS)/relapse-free survival time (RFS), and CD169 expression. The CD169^high^ group showed greater overall survival as compared to the CD169^low^ group; the 5-year CSS was 85.25% in the CD169^high^ group and 72.46% in the CD169^low^ group (*p* = 0.004; **Figure 3A**). In addition, a high density of CD8^+^ TILs was associated with a greater CSS; the 5-year CSS was 93.60% in the CD8^high^ group and 65.36% in the CD8^low^ group (*p* < 0.001; **Figure 3C**). High CD169 expression and a high density of CD8^+^ T cells were independent prognostic factors in a multivariate analysis respectively (**Table 2**; **Figure 3E**). A high density of CD8^+^ TILs was also associated with a greater RFS; the 5-year RFS was 95.61% in the CD8^high^ group and 68.03% in the CD8^low^ group (*p* < 0.001; **Figure 3D**), However, the CD169^high^ group did not show a greater RFS compared to the CD169^low^ group; the 5-year RFS was 86.47% in CD169^high^ and 77.71% in CD169^high^ (*p* = 0.112, **Figure 3B**) groups, respectively. Additionally, in multivariate analysis, a high density of CD8^+^ T cells was an independent prognostic factors, but high CD169 expression was not an independent prognostic factor (**Table 3**; **Figure 3F**).

**Table 1:** Clinicopathological features and CD169^+^/CD68^+^ ratio in lymph node sinus macrophages (LySMs) from 294 patients with gastric cancer.

Clinicopathological feature	n	CD169^+^cells/CD68^+^cells in LySMs		
		<0.65	≥0.65	P
Age				
< 70 (y)	147	62	85	0.198
≥ 70 (y)	147	73	74	
Sex				
Female	109	49	60	0.799
Male	185	86	99	
Histology				
Intestinal type	142	64	78	0.778
Diffuse type	152	71	81	
Depth of invasion				
m, sm	135	66	69	0.346
mp, ss, se, si	159	69	90	
LN metastasis				
Negative	175	80	95	0.932
Positive	119	55	64	
Distant metastasis				
Negative	270	123	147	0.675
Positive	24	12	12	
Lymphatic invasion				
Negative	92	45	47	0.487
Positive	202	90	112	
Vascular invasion				
Negative	141	63	78	0.683
Positive	153	72	81	
Tumor-stroma ratio				
Low	202	96	106	0.413
High	92	39	53	
CD8^+^cells/mm^2^ in tumor				
< 287	147	90	57	<0.001
≥ 287	147	45	102	

LySMs, lymph node sinus macrophages; m, mucosa; sm, submucosa; mp, muscularis propria; ss, subserosa; se, serosa exposure; si, serosa invasion; LN, lymph node.

**Table 2:** Univariate and multivariate Cox regression analysis of potential prognostic factors for cancer specific survival in patients with total gastric cancer (n = 294) and advanced gastric cancer (n = 159).

	Clinicopathological feature	n	Univariate analysis			Multivariate analysis		
			HR	(95%CI)	p	HR	(95%CI)	p
Total gastric cancer	Age							
	<70 (y)	147	1.00					
	≥70 (y)	147	1.02	(0.64 - 1.64)	0.930			
	Sex							
	Female	109	1.00					
	Male	185	1.70	(1.00 - 2.89)	0.050			
	Histology							
	Intestinal type	142	1.00					
	Diffuse type	152	1.20	(0.74 - 1.93)	0.467			
	Depth of invasion							
	m, sm	135	1.00					
	mp, ss, se, si	159	17.06	(6.21 - 46.90)	<0.001	3.60	(1.23 - 10.56)	0.019
	LN metastasis							
	Negative	175	1.00					
	Positive	119	10.96	(5.39 - 19.67)	<0.001	3.08	(1.54 - 6.16)	0.002
	Distant metastasis							
	Negative	270	1.00					
	Positive	24	22.95	(12.90 - 40.84)	<0.001	5.70	(3.02 - 10.76)	<0.001
	Lymphatic invasion							
	Negative	92	1.00					
	Positive	202	40.88	(5.14 - 325.30)	<0.001	4.87×10^4^	(0.00 - 5.65×10^102^)	0.925
	Vascular invasion							
	Negative	141	1.00					
	Positive	153	6.52	(3.33 - 12.77)	<0.001	1.01	(0.49 - 2.09)	0.984
	Tumor-stroma ratio							
	Low	202	1.00					
	High	92	6.88	(4.08 - 11.62)	<0.001	2.29	(1.30 - 4.02)	0.004
	CD8^+^cells/mm^2^ in tumor							
	<287	147	1.00					
	≥287	147	0.17	(0.09 - 0.32)	<0.001	0.37	(0.19 - 0.74)	0.005
	CD169/CD68 ratio in LySMs							
	<0.65	135	1.00					
	≥0.65	159	0.49	(0.30 - 0.80)	0.004	0.41	(0.25 - 0.69)	<0.001
Advanced gastric cancer	Age							
	<70 (y)	80	1.00					
	≥70 (y)	79	0.94	(0.58 - 1.54)	0.807			
	Sex							
	Female	54	1.00					
	Male	105	1.62	(0.93 - 2.83)	0.089			
	Histology							
	Intestinal type	77	1.00					
	Diffuse type	82	1.41	(0.86 - 2.32)	0.175			
	Depth of invasion							
	mp, ss	121	1.00					
	se, si	38	7.64	(4.56 - 12.81)	<0.001	2.86	(1.44 - 5.720)	0.003
	LN metastasis							
	Negative	57	1.00					
	Positive	102	4.21	(2.14 - 8.29)	<0.001	3.40	(1.64 - 7.08)	0.001
	Distant metastasis							
	Negative	135	1.00					
	Positive	24	12.61	(7.00 - 22.70)	<0.001	2.91	(1.32 - 6.43)	0.008
	Lymphatic invasion							
	Negative	4	1.00					
	Positive	155	21.10	(0.04 - 1.15×10^4^)	0.343			
	Vascular invasion							
	Negative	32	1.00					
	Positive	127	1.78	(0.88 - 3.61)	0.108			
	Tumor-stroma ratio							
	Low	84	1.00					
	High	75	4.84	(0.12 - 0.42)	<0.001	2.54	(1.37 - 4.68)	0.003
	CD8^+^cells/mm^2^ in tumor							
	<287	92	1.00					
	≥287	67	0.22	(0.12 - 0.42)	<0.001	0.42	(0.20 - 0.84)	0.015
	CD169/CD68 ratio in LySMs							
	<0.65	69	1.00					
	≥0.65	90	0.36	(0.22 - 0.60)	<0.001	0.38	(0.22 - 0.66)	<0.001

HR. hazard ratio; CI, confidence interval; m, mucosa; sm, submucosa; mp, muscularis propria; ss, subserosa; se, serosa exposure; si, serosa invasion; LN, lymph node; LySMs, lymph node sinus macrophages.

**Table 3:** Univariate and multivariate Cox regression analysis of potential prognostic factors for relapse free survival in patients with total gastric cancer (n = 270) and advanced cancer (n = 135).

	Clinicopathological feature	n	Univariate analysis			Multivariate analysis		
			HR	(95%CI)	p	HR	(95%CI)	p
Total gastric cancer	Age							
	<70 (y)	139	1.00					
	≥70 (y)	131	0.74	(0.42 - 1.32)	0.308			
	Sex							
	Female	101	1.00					
	Male	169	1.83	(0.97 - 3.46)	0.061			
	Histology							
	Intestinal type	134	1.00					
	Diffuse type	136	0.84	(0.48 - 1.48)	0.550			
	Depth of invasion							
	m, sm	135	1.00					
	mp, ss, se, si	135	13.33	(4.79 - 37.09)	<0.001	3.14	(1.07 - 9.23)	0.037
	LN metastasis							
	Negative	172	1.00					
	Positive	98	9.88	(4.79 - 20.38)	<0.001	3.85	(1.82 - 8.15)	<0.001
	Distant metastasis							
	Negative	262	1.00					
	Positive	8	13.90	(6.05 - 31.93)	<0.001	2.34	(0.99 - 5.57)	0.054
	Lymphatic invasion							
	Negative	92	1.00					
	Positive	178	42.94	(3.87 - 4.77×10^2^)	0.002	2.66×10^4^	(0.00 - 2.17×10^90^)	0.920
	Vascular invasion							
	Negative	138	1.00					
	Positive	132	6.19	(2.90 - 13.21)	<0.001	1.12	(0.53 - 2.69)	0.660
	Tumor-stroma ratio							
	Low	196	1.00					
	High	74	5.63	(3.14 - 10.07)	<0.001	2.22	(1.21 - 4.07)	0.010
	CD8^+^cells/mm2 in tumor							
	<287	127	1.00					
	≥287	143	0.17	(0.08 - 0.17)	<0.001	0.21	(0.10 - 0.45)	<0.001
	CD169^+^/CD68^+^cells in RLNs							
	<0.65	119	1.00					
	≥0.65	151	0.64	(0.36 - 1.12)	0.115			
Advanced gastric cancer	Age							
	<70 (y)	72	1.00					
	≥70 (y)	63	0.65	(0.35 - 1.18)	0.156			
	Sex							
	Female	46	1.00					
	Male	89	1.82	(0.92 - 3.58)	0.086			
	Histology							
	Intestinal type	69	1.00					
	Diffuse type	66	1.00	(0.56 - 1.80)	0.991			
	Depth of invasion							
	mp, ss	116	1.00					
	se, si	19	5.53	(2.93 - 10.42)	<0.001	3.64	(1.64 - 8.11)	0.002
	LN metastasis							
	Negative	54	1.00					
	Positive	81	4.03	(1.87 - 8.67)	<0.001	4.97	(2.19 - 11.27)	<0.001
	Distant metastasis							
	Negative	127	1.00					
	Positive	8	7.00	(3.03 - 16.18)	<0.001	1.46	(0.48 - 4.46)	0.507
	Lymphatic invasion							
	Negative	4	1.00					
	Positive	131	21.24	(0.02 - 2.38×10^4^)	0.394			
	Vascular invasion							
	Negative	29	1.00					
	Positive	106	1.62	(0.72 - 3.63)	0.240			
	Tumor-stroma ratio							
	Low	78	1.00					
	High	57	4.15	(2.20 - 7.82)	<0.001	2.03	(1.01 - 4.10)	0.048
	CD8^+^cells/mm in tumor							
	<287	72	1.00					
	≥287	63	0.21	(0.10 - 0.44)	<0.001	0.24	(0.10 - 0.53)	<0.001
	CD169^+^/CD68^+^cells in RLNs							
	<0.65	53	1.00					
	≥0.65	82	0.43	(0.24 - 0.78)	0.005	0.37	(0.19 - 0.72)	0.003

HR. hazard ratio; CI, confidence interval; m, mucosa; sm, submucosa; mp, muscularis propria; ss, subserosa; se, serosa exposure; si, serosa invasion; LN, lymph node; LySMs, lymph node sinus macrophages.

### A Significant Association Between CD169 Expression in LySMs and Clinical Course in Advanced Gastric Cancer Cases

**Figure 4:**
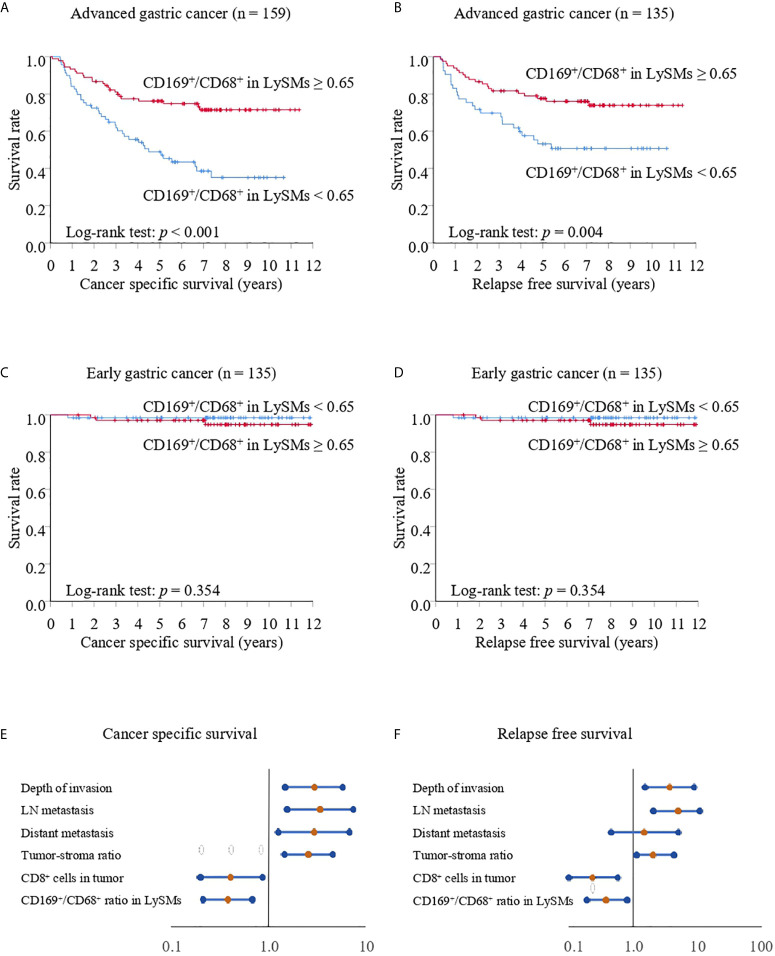
Kaplan-Meier cancer-specific survival and relapse-free survival curves for patients with advanced or early gastric cancer. For all Kaplan-Meier curves, the patients were divided into two groups according to their CD169/CD68 ratio: < 0.65 was defined as low and ≥ 0.65 was defined as high. **(A)** Cancer-specific survival (CSS) curves for patients with advanced gastric cancer. **(B)** Relapse-free survival (RFS) curves for patients with advanced gastric cancer. **(C)** CSS curves for patients with early gastric cancer. **(D)** RFS curves for patients with early gastric cancer. **(E)** Forest plot analysis of CSS in advanced gastric cancer. **(F)** Forest plot analysis of RFS in advanced gastric cancer.

We divided all cases into two groups: early and advanced gastric cancer cases. The CD169^high^ group showed greater CSS and RFS as compared to the CD169^low^ group. The 5-year CSS was 76.19% for the CD169^high^ group, and 48.92% for the CD169^low^ group (*p* < 0.001; **Figure 4A**). The 5-year RFS was 77.58% for the CD169^high^ group and 53.16% for the CD169^low^ group (*p* = 0.004; **Figure 4B**) in advanced gastric cancer. In addition, high CD169 expression was an independent prognostic factor in multivariate analysis (**Tables 2**, **3**, **Figures 4E, F**). However, in early gastric cancer, a significant difference between the ratio of macrophages and prognosis was not noted (**Figures 4C, D**). The 5-year CSS was 97.03% for the CD169^high^ group and 98.48% for the CD169^low^ group (*p* = 0.354; **Figure 4C**). The 5-year RFS was 98.48% for the CD169^high^ group and 97.04% for the CD169^low^ group (*p* = 0.354; **Figure 4D**).

### Significant Association Between CD169 Expression in LySMs and Clinical Course Was Not Dependent on Histological Subtype, Distant Metastasis, Etc.

**Figure 5:**
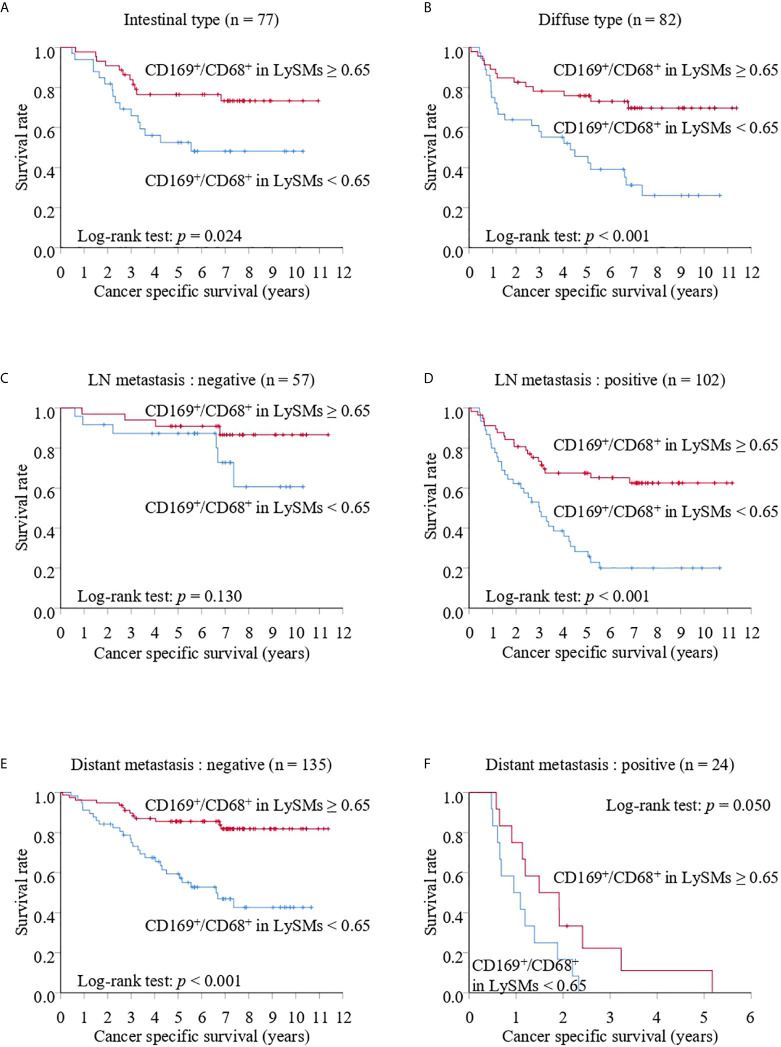
Kaplan-Meier cancer-specific survival (CSS) curves of CD169^+^/CD68^+^ ratios in lymph node sinus macrophages (LySMs) of advanced gastric cancer patients with various tumor subtypes: intestinal type **(A)** or diffuse type **(B)**; lymph node (LN) metastasis negative **(C)** and positive **(D)**; distant metastasis negative **(E)** and positive **(F)**.

**Figure 6:**
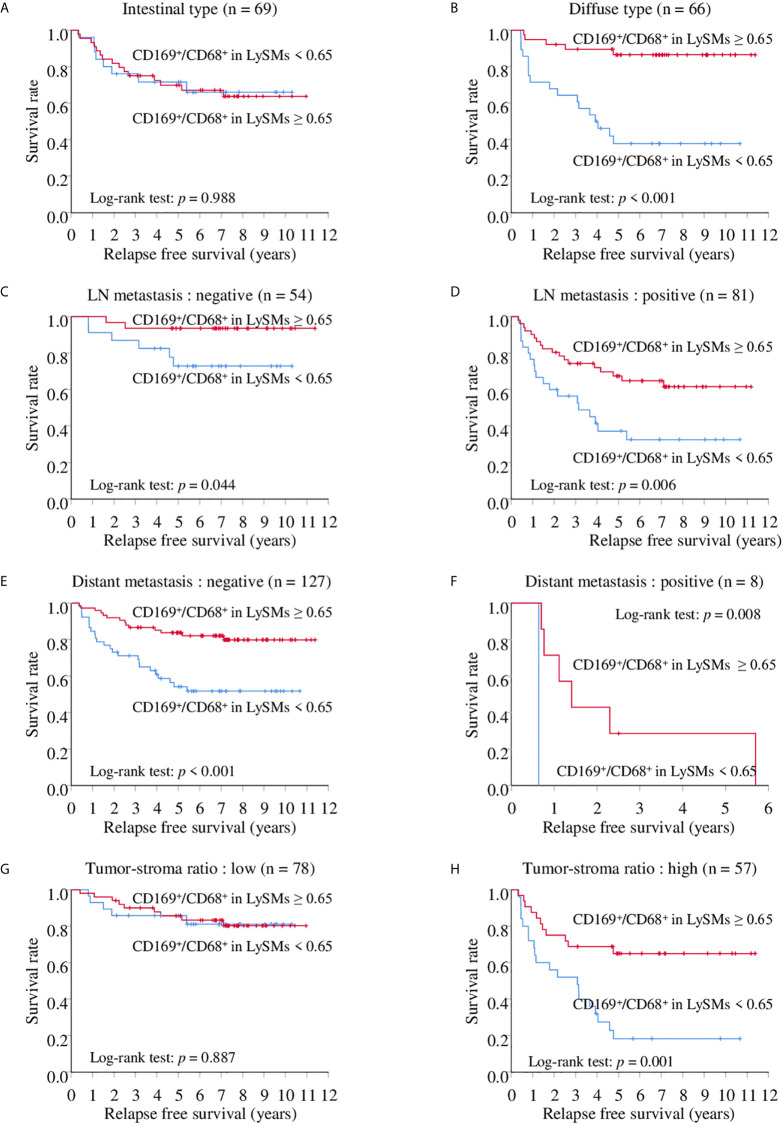
Kaplan-Meier relapse free survival (RFS) curves of CD169^+^/CD68^+^ ratios in lymph node sinus macrophages (LySMs) of advanced gastric cancer patients with various tumor subtypes: intestinal type **(A)** and diffuse type **(B)**; lymph node (LN) metastasis negative **(C)** and positive **(D)**; distant metastasis negative **(E)** and positive **(F)**, tumor-stroma ratio; low **(G)** and high **(H)**.

Because we found a more significant association between CD169 expression in LySM and a clinical course in advanced gastric cancer, we performed a prognostic study of advanced gastric cancer cases only. The CD169^high^ group showed better cancer specific survival in advanced gastric cancer with various subgroups, such as histology, and LN metastasis, in Kaplan-Meier analysis. The 5-year CSS was 76.49% for the CD169^high^ group and 52.54% for the CD169^low^ group for the intestinal type group (*p* = 0.024; **Figure 5A**), and 75.85% in the CD169^high^ group and 45.60% in the CD169^low^ group for the diffuse type group (*p* < 0.001; **Figure 5B**). The 5-year CSS was 67.48% for the CD169^high^ group and 28.27% for the CD169^low^ group for the LN metastasis-positive group (*p* < 0.001; **Figure 5D**); however, no significant difference was seen in cases without LN metastasis (**Figure 5C**). The 5-year CSS was 85.59% for the CD169^high^ group and 59.35% for the CD169^low^ group in a group without distant metastasis (*p* < 0.001; **Figure 5E**). Although a significant difference in cases with distant metastasis was not observed, the CD169^high^ group tended to have a more favorable prognosis. The 5-year CSS was 11.11% for the CD169^high^ group and 0% for the CD169^low^ group (*p* = 0.050, **Figure 5F**). With regards to the RFS, a similar observation that high CD169 expression was associated with a more favorable clinical course tended to be seen. A significant correlation was seen in the diffuse type group but not in the intestinal type group (**Figures 6A, B**). A significant association between high CD169 expression and a more favorable clinical course was seen in a group, with or without LN metastasis, or with or without distant metastasis (**Figures 6C–F**).

### Tumor-Stroma Ratio (TSR) Affected the Correlation Between CD169 Expression in LySMs and the Density of TILs

**Figure 7:**
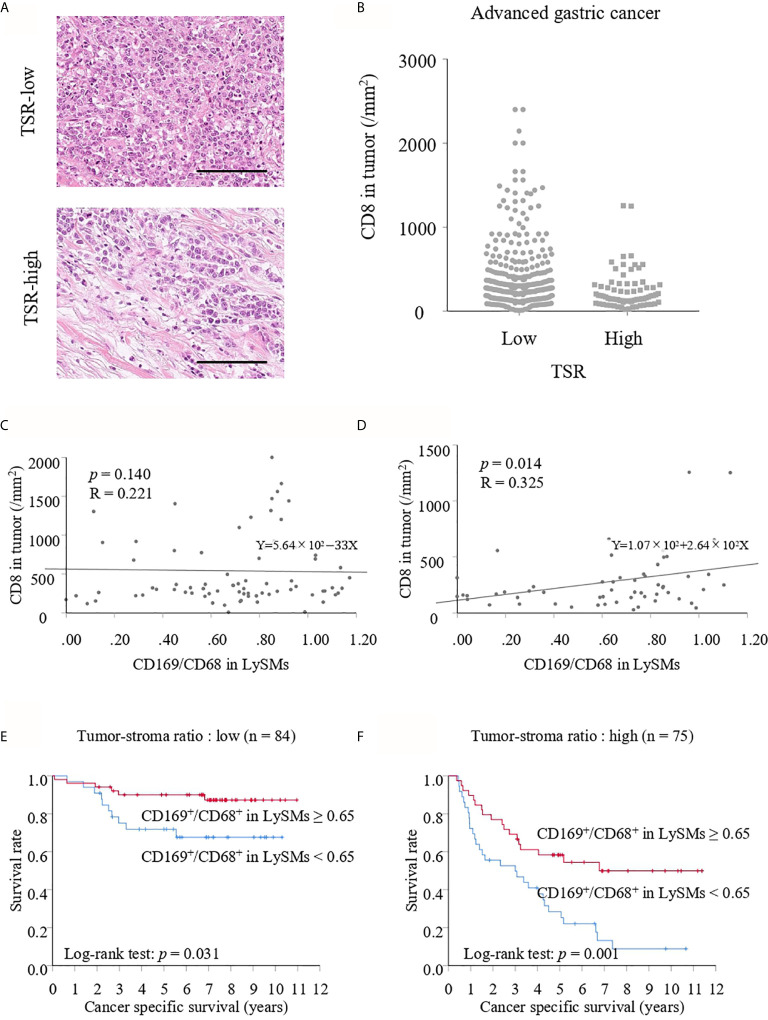
**(A)** Hematoxylin and eosin staining of tumor-stroma ratio (TSR)- low and high. Scale bar = 100 μm. **(B)** Number of CD8^+^ T cells in TSR-low or high advanced cancer tissues. Correlation between the number of CD8^+^ T cells in TSR-low or high tumor tissues and the CD169/CD68 ratio in lymph node sinus macrophages (LySMs) were tested by Spearman’s correlation test. Scatter plot in TSR-low advanced gastric cancer cases **(C)** and in TSR-high advanced gastric cancer cases **(D)** were shown. **(E)** Kaplan-Meier cancer-specific survival (CSS) curves in patients with TSR-low advanced gastric cancer. **(F)** Kaplan-Meier CSS curves in patients with TSR-high advanced gastric cancer.

Because the TSR is a recognized prognostic factor for various solid tumors, we divided advanced gastric cancer into two groups: low and high TSR (**Figure 7A**) as described in the Materials and Methods section. Interestingly, the density of TILs was lower in the high compared with low TSR group (**Figure 7B**). A correlation between CD169 expression and CD8-positive T lymphocytes in the low TSR group was not noted (*p* = 0.140; **Figure 7C**), whereas CD169 expression was positively correlated with the density of CD8-positive T lymphocytes in the high TSR group (R = 0.325, *p* = 0.014; **Figure 7D**). The CD169^high^ group showed greater CSS as compared to the CD169^low^ group for both low and high TSR groups. The 5-year CSS was 90.00% in the CD169^high^ group and 71.84% in the CD169^low^ group for the low TSR group (*p* = 0.031; **Figure 7E**), and 58.33% for the CD169^high^ group and 28.34% for the CD169^low^ group for the high TSR group (*p* = 0.001; **Figure 7F**). Although, a significant difference in the low TSR group in RFS was not observed (*p* = 0.887; **Figure 6G**), the CD169^high^ group showed a superior RFS in high TSR group (*p* < 0.001; **Figure 6H**).

## Discussion

In the present study, we found that a high CD169 expression level was associated with a more favorable overall survival. The observation that CD169 expression was positively associated with the density of CD8-positive cytotoxic T cells (TILs) indicated a significant association between CD169 expression in LySMs and anti-cancer immune responses. These findings are consistent with our previous research findings in colorectal cancer, melanoma, esophageal cancer, and bladder cancer (21, 28–30). Since CD169 overexpression has been suggested to be linked to interferon production (21), this might indicate an inflammatory reaction in lymph nodes.

**Figure 8:**
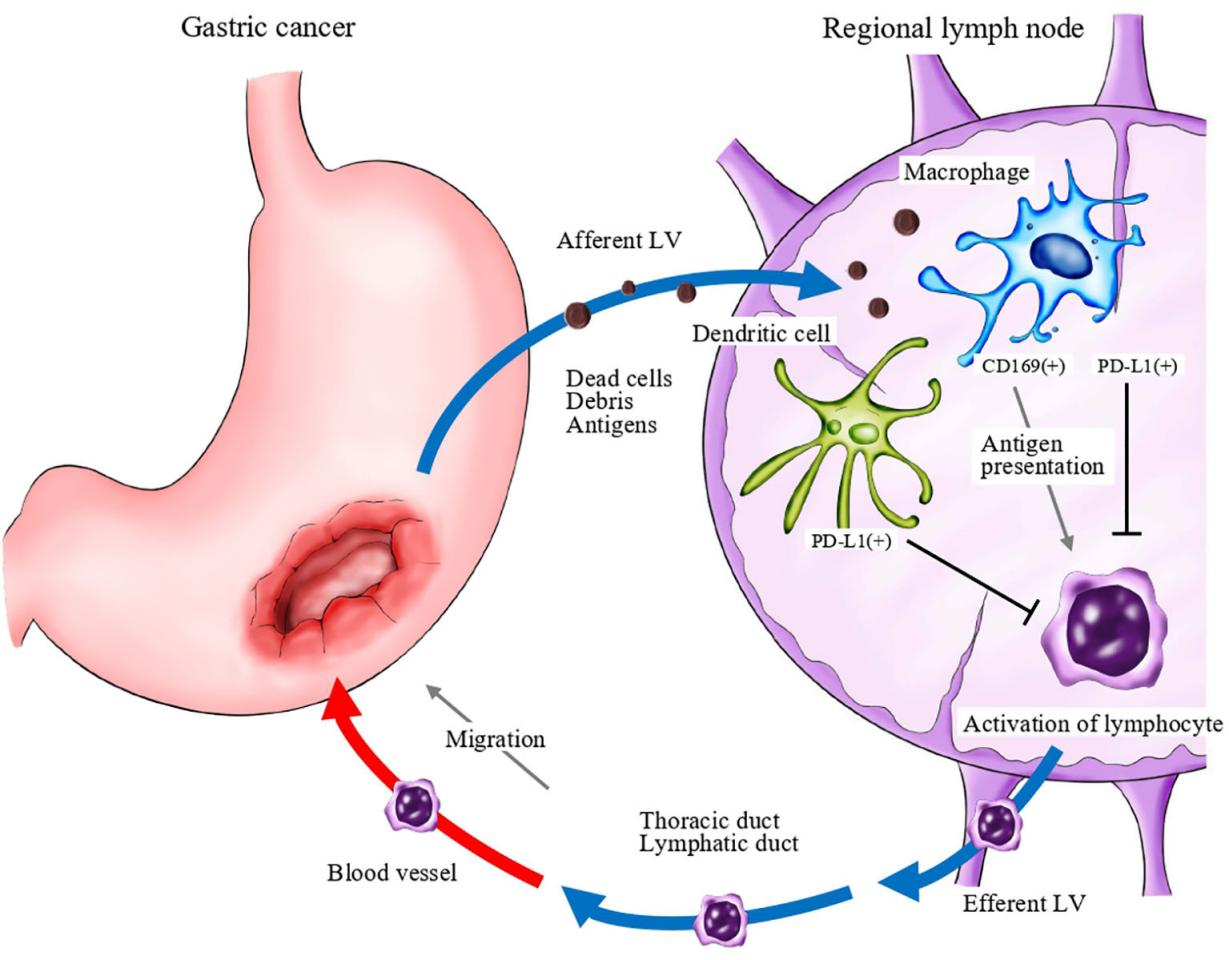
A schema of the anti-cancer immune response associated with regional lymph nodes. Dead cells and debris, including tumor-specific antigens, drain into lymph node sinuses *via* lymphatic vessels (LV). Antigen-presenting cells, such as macrophages and dendritic cells, capture and engulf these antigens and activate antigen-reactive T lymphocytes. Programmed death ligand 1 (PD-L1; and potentially PD-L2) expressed on antigen-presenting cells may negatively regulate the activation of T lymphocytes.

Immune checkpoint blockade targeting programmed death 1 (PD-1)/programmed death ligand 1 (PD-L1) and cytotoxic T lymphocyte-associated protein 4 (CTLA-4) has become a promising approach for anti-cancer immunotherapy (31). The ATTRACTION-2 study was conducted in patients with gastric cancer who had become resistant to second- and third-line treatments. A significant difference in overall survival (OS) was observed between nivolumab and placebo groups (32). PD-1 ligands are expressed not only on cancer cells, but also on immune cells. Among immune cells, antigen-presenting cells, such as macrophages and dendritic cells, express high levels of PD-1 ligands (33, 34). Recently, it was reported that PD-L1 expression was significantly elevated in myeloid cells in the lymph nodes of cancer-bearing mice, and anti-PD-1 therapy induced T cell activation and proliferation in the lymph nodes (35). The study also demonstrated that resection of draining lymph nodes completely abrogated anti-tumor immune responses that were induced by PD-1/PD-L1 blockade therapy. These data indicated that PD-1/PD-L1 signals work as a negative regulator at regional lymph nodes, and that lymph nodes are pivotal sites for the induction of anti-tumor T cells.These findings suggest that CD169 positive macrophages may involve the mechanisms that are described in **Figure 8**.

Interestingly, the CD169^high^ group had greater CSS than the CD169^low^ group in the TSR high group. Because the stromal component of tumors has been proven to have a significant impact on tumor development (36), it has been recognized as a potential prognostic factor for various solid tumors (37). Cancer-associated fibroblasts (CAFs), which produce a desmoplastic stromal component, are thought to be important in considering the TSR (38). It is possible that CD169-positive cells have some effect on CAFs. The CD169^high^ group also had a significantly greater RFS in patients with gastric cancer in a high recurrence group, including diffuse type, LN metastasis, distant metastasis, and high TSR, suggesting a better prognosis because of reduced recurrence.

Regardless of CD169 expression in LySMs, significant high density of CD8-lymphocytes was counted in some cases. Some of these cases were suggesting medullary carcinoma or gastric carcinoma with lymphoid stroma. It is speculated that these cases could potentially contain EBV-positive or MSI-high gastric carcinoma, although these were not sufficiently investigated in this study.

In conclusion, CD169 overexpression in LySMs is a predictor of a more favorable clinical course in association with anti-cancer immune responses. In addition, CD169 expression in LySMs as well as the density of TILs were identified as independent prognostic factors in the multivariate analysis. The evaluation of CD169 in RLNs might enable us to predict the anti-cancer immune responses in gastric cancer.

## Data Availability Statement

The original contributions presented in the study are included in the article/supplementary material. Further inquiries can be directed to the corresponding author.

## Ethics Statement

The studies involving human participants were reviewed and approved by Ethics Committee of Medical Research, University of Occupational and Environmental Health, Japan. The patients/participants provided their written informed consent to participate in this study.

## Author Contributions

KK, KO and YK designed the study. KK and YK executed experimental work. KK, TT, KO, SS, YK and TN analyzed and interpreted data. KK, MS and YK performed the statistical analysis. KK drafted the manuscript. All authors contributed to the article and approved the submitted version.

## Funding

This work was supported by grants from the Ministry of Education, Culture, Sports, Science and Technology of Japan (Nos. 18K06991) and Takeda Science Foundation.

## Conflict of Interest

The authors declare that the research was conducted in the absence of any commercial or financial relationships that could be construed as a potential conflict of interest.
